# Unraveling the Endocannabinoid System: Exploring Its Therapeutic Potential in Autism Spectrum Disorder

**DOI:** 10.1007/s12017-024-08781-6

**Published:** 2024-05-14

**Authors:** Ankit Jana, Arnab Nath, Palash Sen, Swikriti Kundu, Badrah S. Alghamdi, Turki S. Abujamel, Muhammad Saboor, Chan Woon-Khiong, Athanasios Alexiou, Marios Papadakis, Mohammad Zubair Alam, Ghulam Md Ashraf

**Affiliations:** 1 Department of Biological Sciences, National University of Singapore, Singapore, 117558 Singapore; 2 Department of Developmental Biology and Genetics, Indian Institute of Science, Bangalore, Karnataka 560012 India; 3 School of Biosciences, Indian Institute of Technology, Kharagpur, West Bengal 721302 India; 4 Siksha Bhavana, Visva-Bharati University, Bolpur, West Bengal 731235 India; 5 Pre-Clinical Research Unit, King Fahd Medical Research Center, King Abdulaziz University, Jeddah, Saudi Arabia; 6 Department of Physiology, Neuroscience Unit, Faculty of Medicine, King Abdulaziz University, Jeddah, Saudi Arabia; 7 Vaccines and Immunotherapy Unit, King Fahd Medical Research Center, King Abdulaziz University, Jeddah, Saudi Arabia; 8 Department of Medical Laboratory Technology, Faculty of Applied Medical Sciences, King Abdulaziz University, Jeddah, Saudi Arabia; 9 Department of Medical Laboratory Sciences, College of Health Sciences, and Research Institute for Medical and Health Sciences, University of Sharjah, P.O. Box 27272, Sharjah, United Arab Emirates; 10 University Centre for Research & Development, Chandigarh University, Chandigarh-Ludhiana Highway, Mohali, Punjab India; 11 Department of Research & Development, Funogen, Athens, Greece; 12 Department of Research & Development, AFNP Med, 1030 Vienna, Austria; 13 Department of Science and Engineering, Novel Global Community Educational Foundation, Hebersham, NSW 2770 Australia; 14 Department of Surgery II, University Hospital Witten-Herdecke, University of Witten-Herdecke, Heusnerstrasse 40, 42283 Wuppertal, Germany; 15 Department of Medical Laboratory Sciences, Faculty of Applied Medical Sciences, King Abdulaziz University, Jeddah, Saudi Arabia

**Keywords:** Autism spectrum disorder, Endocannabinoid system, Anandamide (AEA), 2-Arachidonoylglycerol (2-AG)

## Abstract

The salient features of autism spectrum disorder (ASD) encompass persistent difficulties in social communication, as well as the presence of restricted and repetitive facets of behavior, hobbies, or pursuits, which are often accompanied with cognitive limitations. Over the past few decades, a sizable number of studies have been conducted to enhance our understanding of the pathophysiology of ASD. Preclinical rat models have proven to be extremely valuable in simulating and analyzing the roles of a wide range of established environmental and genetic factors. Recent research has also demonstrated the significant involvement of the endocannabinoid system (ECS) in the pathogenesis of several neuropsychiatric diseases, including ASD. In fact, the ECS has the potential to regulate a multitude of metabolic and cellular pathways associated with autism, including the immune system. Moreover, the ECS has emerged as a promising target for intervention with high predictive validity. Particularly noteworthy are resent preclinical studies in rodents, which describe the onset of ASD-like symptoms after various genetic or pharmacological interventions targeting the ECS, providing encouraging evidence for further exploration in this area.

## Introduction

Autism spectrum disorder (ASD) refers to a group of neurodevelopmental disorders that involve significant difficulties in communication and social interaction, as well as the presence of restricted, repetitive, and stereotyped patterns of behavior. Along with these defining features, ASD is commonly associated with a rage of comorbidities, including aggression, hyperactivity, seizures, depression, sleep disturbances, gastrointestinal problems, and immunological malfunction. The global incidence rate of ASD is estimated to be approximately 1%, with a male-to-female ratio of approximately 3:1 (Werling & Geschwind, 2013). Despite its significant public health impact and high prevalence rates, the pathogenesis of ASD remains poorly understood, in large part due to ASD’s complicated genetic and environmental interactions. Recent literature suggests that ASD is characterized by impairments in synaptic function, which are believed to contribute to the core symptoms of the disorder. Consequent to the observed synaptic impairments in ASD, the endocannabinoid system (ECS) has gained significant attention as a potential contributor to the initiation and/or progression of the disorder. This is because the ECS has the ability to modulate a variety of synaptic mechanisms, including neurotransmission, synaptic currents, inhibition (E/I balance), and neuroplasticity. Moreover, the ECS has been implicated in several processes that are frequently affected in individuals with ASD, such as social communication, motor control, repetitive behaviors, emotional control, as well as learning, and memory (Zou et al., 2021).

ECS comprises cannabinoid receptors CB1, found as a neuronal target of the psychoactive ingredient of *Cannabis sativa*, 9-tetrahydrocannabinol (THC), and CB2, and their endogenous lipid ligands, i.e., the endocannabinoids (eCBs). These eCBS include anandamide (AEA) and 2-arachidonoylglycerol (2-AG), which are synthesized on demand and function as retrograde neurotransmitters (Pascucci et al., 2020; Su et al., 2021). It is noteworthy that the ECS provides a critical link between the immune system and the central nervous system (CNS). CB2 receptors are primarily found on immune cells and modulate the immune system, whereas CB1 receptors are found abundantly in the CNS (particularly in the hippocampus, cerebral cortex, basal ganglia, and cerebellum), and peripheral nervous system (PNS).

The involvement of the ECS in ASD extends beyond its influence on synaptic function and neuroplasticity. Indeed, emotional and behavioral responses to social and environmental stimuli as well as modulation of learning, memory, seizure susceptibility, and circadian rhythm, are also thought to be regulated by the ECS in ASD (Marco & Laviola, 2012; Marsicano & Lutz, 2006; Rubino et al., 2008; Trezza & Vanderschuren, 2008; Trezza et al., 2012). This highlights the broad impact that the ECS has on various physiological and behavioral processes that are disrupted in individuals with ASD. In addition to preclinical studies, human neuroimaging research has also uncovered the relationships between polymorphisms in the CB1 receptor gene, *CNR1*, and social reward sensitivity, suggesting that variations in CB1 receptors could contribute to ASD-related irregularities in social reward processing. Furthermore, postmortem analysis of the brains diagnosed with ASD has revealed lower CB1 receptor expression, adding further support to the notion that the ECS plays a key role in the pathogenesis of ASD (Aishworiya et al., 2022; Baron-Cohen, 2004; Chakrabarti et al., 2006; Domschke et al., 2008). Regardless of the fact that these data imply the involvement of the ECS in ASD, there is still a dearth of research exploring the role of ECS in ASD, and our knowledge of the EC signaling in ASD remains limited.

This review focuses on studies investigating ECS alterations and the effects of pharmacological modulation of the ECS in animal models of ASD. In addition, the potential of the ECS as a therapeutic target for ASD is discussed.

## Genetic Model of ASD and ECS

Variations in the genes encoding CB1 receptors have a significant impact on social behavior in individuals. The observed reduction in CB1 receptors levels among ASD patients suggests a direct association between them. However, studies have shown that CB1 receptor ligand AEA is present in relatively lower amounts in ASD children, whereas the 2-AG level remains unchanged (Pietropaolo et al., 2020).

The CB2 receptor was first identified in 1993 through cDNA-based polymerase chain reaction (PCR) clone of the human promyelocytic leukemic line HL60, using degenerative primers (Munro et al., 1993). CB2 receptors belong to the G-protein-coupled receptor family and are composed of an internal C-terminal, three extracellular and three intracellular loops, seven transmembrane domains, and an external N-terminal. CB2 receptors show approximately 44% amino acid sequence similarity to CB1 receptors with hydrophobic domains 1, 2, 5, 6, 7, and the extracellular domain of both receptors sharing at least 50% similarity in amino acid sequence (Matsuda, 1997). The *CNR2* gene is highly conserved across different taxa. The human *CNR2* gene consists of a single translated exon flanked by 5' and 3' untranslated regions and a single untranslated exon (Sipe et al., 2005). However, transcriptional products of mammalian CB2 receptor genes (CNR2) vary among species. The mouse (23 kb) and rat (20 kb) CB2 receptor genes are almost four times shorter than the human CB2 receptor gene (90 kb). The mouse CB2 receptor gene is transcribed into two mRNAs from three exons, whereas the rat CB2 receptor gene can be spliced into four mRNAs from three exons (Cording & Bateup, 2023; Onaivi et al., 2013). Compared to human CB2 receptors, the amino acid sequence homology is lower between human and mouse CB2 receptors (82%) than between human and rat CB2 receptors (93%). Although polymorphism of the *CNR2* gene is not well studied, but it may be associated with depression in humans. However, Karsak et al. reported that *CNR2* gene polymorphism correlates with osteoporosis and other autoimmune diseases (Karsak et al., 2005).

Further studies in the Japanese population showed that Q63R polymorphism of the *CNR2* gene is linked with alcoholism (Ishiguro et al., 2007) and depression (Onaivi et al., 2013). *CNR2* gene expression in peripheral immune cells prevents inflammation and neuronal damage and exerts specific changes in the central nervous system. Activated glial cells, NK cells, and monocytes show the highest levels of CB2 receptors indicating that CB2 receptors may be a key player in cytokines release and immune cell migration during different pathophysiological conditions. CB2 receptors have been found to be upregulated during inflammation in other brain-associated cells and have been shown to play a vital role in reducing depression in rodents (Morcuende et. al 2022, Garcia-Gutierrezet. al 2018, Onaivi et. al 2008). Furthermore, the  expression level of the *CNR2* gene has been shown to increase in the hippocampus of offspring exposed to VPA (Onaivi, 2006). The evidence presented suggests that overexpression of *CNR2* gene could be a potential therapeutic approach for treating inflammation and depression in autistic children. *CNR2* and *FAAH* genes are closely linked in mice and humans. It has been shown that anandamide-deactivating enzyme FAAH inhibition can ameliorate social disabilities in ASD-related models BTBR and *fmr1*^*−/−*^ mice (Wei et al., 2016). It is likely that bidirectional modulation of *CNR2* and *FAAH* genes would likely increase social interaction and reduce anxiety and neuroinflammatory responses in autistic children.

**Fig. 1:**
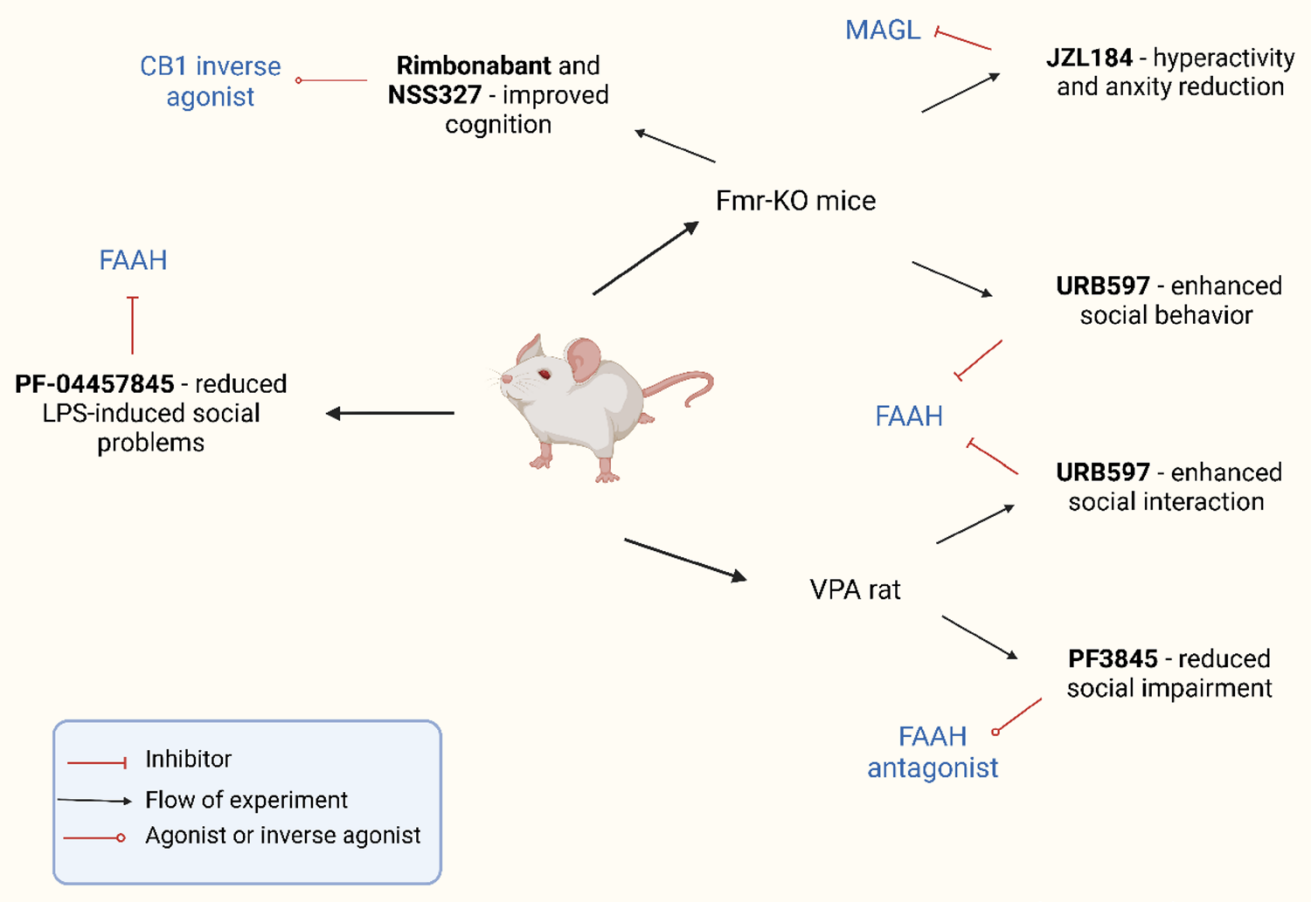
Simplified representation of the endocannabinoid (EC) system upon nerve stimulus. The endocannabinoids (AEA and 2-AG) trigger the CB1 receptors of presynaptic neurons. 2-AG is generated through hydrolysis of DAG by the DAGLα and DAGLβ enzymes, whereas AEA is synthesized through the action of NAPE-PLD enzyme. Membrane depolarization or nerve stimulation elevates the intracellular Ca^2+^ level that induces the 2-AG and AEA production in postsynaptic neurons. Retrograde attachment of AEA and 2-AG to CB1 receptors initiates downstream pathways that lowers Ca^2+^, reduces neurotransmitter release and leads to endocannabinoid degradation via MAGL and FAAH. Rimonabant and NESS3027 are two potential inhibitors of CB1 receptors. CB2 receptors are preferentially found in immune cells and reduce IL-1 expression. AM630 blocks CB2 receptors

In addition, Fragile X Syndrome (FXS) is one of the significant monogenetic reasons for ASD. FXS occurs due to mutation in the *Fmr1* gene on the X chromosome, which leads to reduction or absence of the FMRP protein (Zou et al., 2021). FMRP is instrumental for the normal development of synapses in the brain, and its absence or reduction can cause various symptoms such as developmental delay, anxiety, intellectual and physical disabilities, and repetitive behaviors among others (Garber et al., 2008). FXS also leads to ASD in at least 30% of cases, and the Fmr1 knockout mouse is considered a model system for FXS (Hagerman et al., 2010; Kazdoba et al., 2014). Patients suffering from FXS have an impaired endocannabinoid signaling system (Zhang & Alger, 2010). Moreover, studies have shown that modulation of either CB1 or CB2 receptors in the Fmr1 knockout mouse can improve some behavioral symptoms associated with ASD (Arnau Busquets-Garcia et al., 2013). JZL184 increases CB1 receptors through the 2-AG signaling pathway, and its application in Fmr1 knockout mouse led to decrease in behavioral abnormalities (Fig. 1) (Arnau Busquets-Garcia et al., 2013; Jung et al., 2012). In addition, the deletion of the *CB1* receptor gene (CN1R) or pharmacological blockage of CB1 receptors resulted in reduced cognitive and seizure-related neurological problems in Fmr1 knockout mouse. However, rimonabant, a promising CB1 targeting drug, has been associated with severe adverse effects (Maria Gomis-González et al., 2016). Modulation of AEA levels may be a potential therapeutic approach. FAAH inhibitors increase the level of AEA, and their application improves cognitive and social behavioral problems in Fmr1 knockout mice (Fig. 1) (Qin et al., 2015). Furthermore, treatment with the CB2 receptor agonist AM630 has shown to ameliorate anxiety and audiogenic seizure behaviors in the Fmr1 knockout mouse model (Fig. 1). Therefore, drugs that can alter the efficiency of CB2 receptors may be a potential therapeutic target to cure ASD-related behavioral traits (Busquets-Garcia et al., 2013).

Neuroligins (NLGNs) are a group of postsynaptic cell adhesion molecules. They control the maturity and function of excitatory and inhibitory synapses in the mammalian brain (Jamain et al., 2008; Südhof, 2008). Mutations of *NLGN3* and *NLGN4* in humans are associated with seizures, X-linked intellectual disability, and autistic behavior. In particular, the *Arg*_*451*_*Cys* (R451C) missense mutation of *NLGN3* has been linked with ASD in humans. Similarly, *NLGN3* mutant mice with the *R*_*451*_*C* mutation show impaired social communication, increased synaptic inhibition in the somatosensory cortex, and excitatory transmission in the hippocampus (Etherton et al., 2011). *NLGN3* mutant mice model not only expresses partial characteristics of ASD condition, but it also provides sufficient information about synaptic gene regulation and ASD (Radyushkin et al., 2009). Studies on NLGN3R451C knock-in and NLGN3 knockout mouse models have shown that disruption of tonic EC signaling mediated by CB1 receptors in the hippocampus and the somatosensory cortex causes autistic behaviors (Zamberletti et al., 2017). Although CB2 receptors do not have a direct role in controlling NLGNs associated ASD-like phenotypes, a combination of drugs modulating both CB1 and CB2 receptors may be a possible pharmacological approach to mitigate ASD-related symptoms. Alteration in CB2 receptors activity and AEA metabolism have been observed in blood monocyte-derived macrophages and peripheral blood mononuclear cells of ASD patients (Siniscalco et al., 2013). This evidence suggests that CB2 and other endocannabinoid signaling compounds may play a critical role in influencing ASD-related symptoms. Nevertheless, zebrafish and humans share similarities in the endocannabinoid pathway (Bailone et al., 2022), including CB1, CB2 receptors, as well as key enzymes of the endocannabinoid system, such as prostaglandin-endoperoxide synthase 2, fatty acid amide hydrolase, and transient receptor potential Cation Channel 1A (Elphick, 2012; Klee et al., 2012; Lam et al., 2006). Studies using the zebrafish model have established that the CB2 inverse agonist JTE‐907 acts as an anxiogenic agent, while the non‐selective CB agonist WIN 55,212 has anxiolytic effects (Hasumi et al., 2020; Prasad et al., 2020).

## Role of ECS in Pathophysiology of ASD

Uncovering the etiopathogenesis of ASD is extremely challenging because this ailment arises from a complex interplay of multiple genetic and environmental factors that act through a multitude of complicated disease mechanisms, such as imbalances between neuronal excitation and inhibition and hypo- and/or hyper-connectivity (Pardo & Eberhart, 2007). As mentioned earlier, the genetics of ASD are extremely diverse, involving hundreds of ASD susceptibility genes (Roux et al., 2019). The intricacy appears to preclude any simple characterization of pathophysiological mechanisms that can explain the interactions and permutative effects of polygenic mutations, as well as the role of environmental impact (McOmish et al., 2014). However, successful understanding of the etiopathology of ASD can immediately lead to the discovery of new therapeutic options that can treat the root cause of ASD. Currently, existing therapeutic interventions for ASD patients only target peripheral symptoms such as anxiety, irritability, aggression, and seizures, and they are treated with anxiolytics, antipsychotics, and anticonvulsants, in that order. These symptom-focused treatments do not address the root cause of ASD, and they are linked with severe side effects that are particularly undesirable in children. To solve the pressing need for better treatments, animal research is focused on identifying new molecular targets for potent interventions by dissecting prevalent ASD etiopathological pathways.

The ECS has demonstrated pathogenetic significance and potential as a novel therapeutic target in our search for shared contributing factors for ASD.

**Fig. 2:**
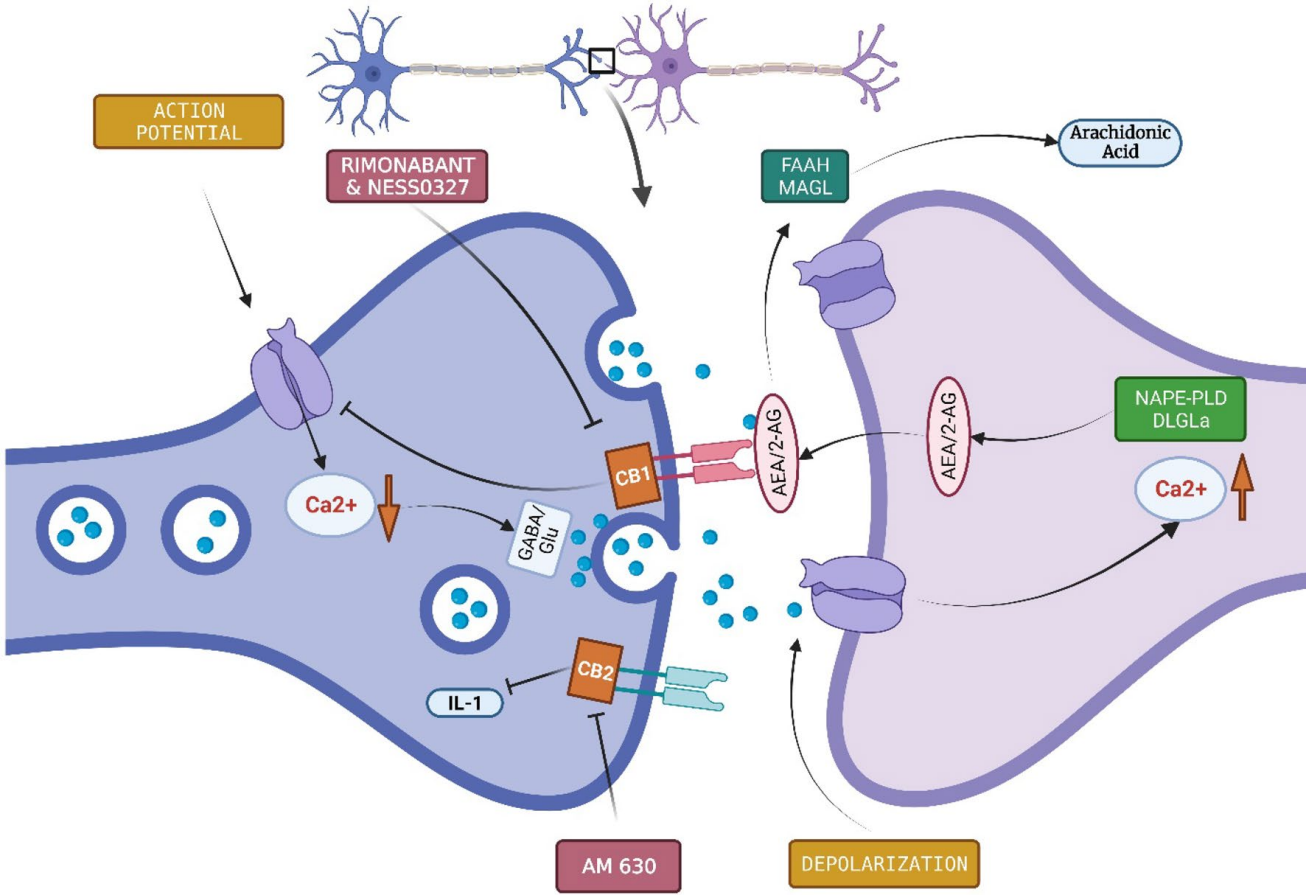
Role of Endocannabinoid System in Pathophysiology of ASD. CB1 and CB2 receptors can work as a potential therapeutic target of ASD. Rimonabant & NESS0327 targets CB1; whereas AM630 targets CB2

Firstly, CB1Rs are extensively expressed in the brain (Mackie, 2005). Second, the ECS has emerged as an essential modulator of neuronal function. Endocannabinoid signaling affects synaptogenesis and neural interconnectedness throughout development. Impairment of these pathways underlies the pathophysiology of autism spectrum disorder. As shown in Fig. 2, CB1 receptors are present presynaptically in both GABAergic and glutamatergic neurons. They are triggered by endogenous ligands such as AEA and 2-AG (Berghuis et al., 2007; FREUND et al., 2003). After membrane depolarization or excitation of metabotropic receptors, endogenous cannabinoids including AEA and 2-AG are generated at the postsynaptic location. This generation is caused by calcium elevation, which can induce plasma membrane lipid reconfiguration. The biosynthetic enzyme N-acyl phosphatidylethanolamine-specific phospholipase D (NAPE-PLD) produces AEA, while diacylglycerol lipase alpha (DAGL-a) produces 2-AG (FREUND et al., 2003). These endogenous cannabinoids retrogradely attach to presynaptic CB1 receptors, activating multiple intracellular pathways that lower intracellular calcium levels and restrict neurotransmitter release. Endogenous cannabinoids are eliminated through a reuptake mechanism. AEA is decomposed by fatty acid amide hydrolase (FAAH), and 2-AG by monoacyl-glycerol lipase (MAGL).

In recent times, behavioral conditions including depression, autism, and schizophrenia have been linked to dopamine abnormalities. Evidence from neurochemistry demonstrates that activation of CB1R expression on GABA neurons in the ventral tegmental area (VTA) reduces GABAergic transmission, which in turn increases dopaminergic neurotransmission in the nucleus accumbens (NAc) (Sperlágh et al., 2009). Clarifying the connection between ECS and DA in ASD may aid in improving our knowledge of the etiopathogenesis of the disorder and may lead to the development of new treatment approaches, as dopamine signaling anomalies have been linked to ASD in both animal models (Pascucci et al., 2020) and autistic individuals (Su et al., 2021).

## Neuroimmunology of ASD and ECS

Microglia and macrophage are considered to be the key immune cells in repairing CNS injuries and infections, as they mediate phagocytosis of pathogens and initiate neuroinflammatory responses by releasing cytokines such as IL-1, IL-6, TNFα, etc. (Janda et al., 2018; Yang et al., 2010). In recent times, neuroimmunologists have been largely focusing on microglia, the resident immune population of brain parenchyma, which is classified as mononuclear phagocytes, including monocyte-derived cells, dendritic cells, peripheral and CNS associated macrophages (Gomez Perdiguero et al., 2013; Prinz et al., 2011). Microglia are derived from a common pool immune progenitors found in the fetal yolk sac, which also give rises to astrocytes and oligodendrocytes (Ginhoux et al., 2010, 2013). After entering the CNS at embryonic day 9.5, microglia arrive in the CNS before astrocytes and even before the commencement of true cortical neurogenesis, which begins at approximately embryonic day 12 (Hughes et al., 2023; Miller & Gauthier, 2007).

### Microglial Involvement and Biology of ASD

Positron emission tomography (PET) and post-mortem analyses have both showed high levels of neuroinflammation and increased microglia activation in the brains of individuals with ASD, indicating the microglial involvement in ASD (Morgan et al., 2010; Vargas et al., 2005). Recently, a distinct microglial signature has been observed from large-scale transcriptomic data analysis from post-mortem cerebral cortex (Suzuki et al., 2013). Changes in synaptic density have also been observed in post-mortem ASD brain tissues (Hutsler & Zhang, 2010), and in ASD mouse models (Comery et al., 1997; Hughes et al., 2023; Tang et al., 2014; Wang et al., 2017). These changes may be due to defects in developmental synaptic pruning (Hansel, 2019). Indeed, current evidence suggests that microglia may contribute to ASD progression through dysregulation of synaptic pruning (Di Marco et al., 2016; Lenz & Nelson, 2018). This hypothesis is supported by the finding that inhibition of microglia autophagy increases synaptic density and reduces sociability in mice (Kim et al., 2017). Studies on the mouse model of Rett syndrome (RTT), a syndromic form of ASD caused by mutations in the methyl-CpG binding protein 2 (MECP-2) encoding gene, provide additional support for the involvement of microglia in ASD pathogenesis (Lombardi et al., 2015). One model of RTT showed that neuronal loss of MECP-2 caused increased synaptic engulfment by microglia in subsequent stages of disease, although microglia themselves did not exhibit any loss (Schafer et al., 2016). This suggests that neuronal loss of MECP-2 alone is sufficient to induce aberrant microglial activity.

In ASD, aggregated alpha-synuclein released from dying dopaminergic neurons activates microglia, leading to high production of proinflammatory cytokines and reactive oxygen species (ROS) which is one of the hallmarks of ASD. Transforming growth factor beta (TGF-β) is one of the factors involved in ASD. Lower levels of TGF-β have been observed in ASD children with high behavioral scores. In addition, macrophage inhibitory factor (MIF), which plays a role in the neural and endocrine systems, is also associated with ASD (Fingerle-Rowson & Bucala, 2001). Two polymorphisms in the promoter region of MIF linked with autism have been observed in genotypic studies of 1000 families (Grigorenko et al., 2008). Differences in NK cell activity have also been observed in ASD patients with some studies showing reduced cytotoxic activity of NK cells in ASD children compared to control. Toll-like receptors (TLRs) expressed by monocytes also act as markers of ASD. TLR-2 and TLR-4 stimulation of monocytes produce proinflammatory cytokines in ASD individuals compared to controls. However, TLR-9 stimulation showed decreased production of proinflammatory cytokines in ASD compared with non-ASD patients, suggesting that monocytes from ASD children have different response in stimulating innate immunity compared with controls. Recent studies on post-mortem brain and spinal cord samples from 11 individual with ASD have revealed high activation of microglia and astroglia, as well as increased levels of cytokines monocyte chemotactic protein-1 (MCP-1) and TGF-beta compared to control (Vargas et al., 2005). After measuring the cytokine levels in brain samples of individuals with ASD in comparison to age and sex-matched non-ASD individuals, there was a significant increase in proinflammatory and Th1 cytokines. These studies provide a clear insight into the immune status of ASD, and due to its anti-inflammatory activity, the ECS can be considered a promising tool for modulating microglial involvement in ASD.

## Microglial-Endocannabinoid Signaling and ASD

Preclinical evidence supporting a role of ECS signaling in ASD comes from studies in rodent models of MIA and neuroinflammation (Hughes et al., 2023; Salloum-Asfar et al., 2023). For example, the production of MIA-based IL-17 in response to the innate immune stimulator polyinosine:polycytidylic acid [poly(I:C)] induces abnormal cortex development and ASD-like sociability deficits in mouse offspring (Gunn et al., 2016). Recent in vivo and in vitro studies suggest that ECS plays an outstanding role in communication between the nervous and immune systems during neuronal damage and inflammation in the CNS. Some studies have reported the proinflammatory role of cannabis in protecting against the activation of microglia with a complex mechanism (Bailone et al., 2022; Killestein et al., 2003; Maestroni, 2004). Moreover, proinflammatory cytokines such as IL-6, IL-12, and TNF-α can cause neuroinflammation and neurodegeneration (Wang et al., 2015). The activation of ECS has been identified as one of the mechanisms that protect against the detrimental effects of these proinflammatory cytokines. During inflammation, 2-AG, which is a ligand of endocannabinoid receptor, is released from various immune cells and induces neuroprotection through several mechanisms by binding to endocannabinoid receptor (Zou & Kumar, 2018). Microglial cells and astrocytes produce 2-AG in response to intracellular Ca^+2^ and glutamate receptor stimulation, which stimulates purinergic P2X7 receptor (Carrier et al., 2004; Hu et al., 2022). The ECS, specially CB2 receptor, mediates T and B lymphocytes proliferation, apoptosis, macrophage-mediated killing of sensitized cells, production of inflammatory cytokines by the inhibiting cyclic AMP/ Protein kinase A (PKA) pathways, migration of B cells, and cytokines induction. Dendritic cells also have the capability to undergo cannabinoid-induced apoptosis due to their immunosuppressive properties (Do et al., 2004; Hu et al., 2022). So, ECS can be a crucial target for ASD therapies due to its anti-inflammatory and immunosuppressive effects. Targeting the ES receptor can lead to side effects like inhibition of learning and memory, hence the ES CB2 receptor, which is present in microglia, is being targeted nowadays. High levels of mRNA and protein of the CB2 receptor have been observed in the blood of autistic children, suggesting its essential role in ASD (Hu et al., 2022; Siniscalco et al., 2013). Various pharmacological molecules can be used to reduce microglia-mediated neurodegeneration and inflammation and beta-amyloid induced neurotoxicity by modulating various ECS receptors like CB1, CB2 and unknown receptors. In the future, we should focus on altered eCB signaling in microglia and the mechanism by which it yields protective and detrimental effects in CNS to provide improved therapies for ASD patients(Kibret et al., 2023).

## CBS as a Potential Therapeutic Target of ASD

Several animal models of ASD have shown variations in ECS functionality through various techniques. The Fmr1-KO mouse model of FXS, a monogenic developmental condition linked to ASD, has been investigated the most regarding the ECS in relation to ASD models. Since FXS also lacks therapies, researchers have also explored the ECS for potential medications. In Fmr1-KO mice, aberrations and imbalances in EC signaling have been observed, indicating that their correction via 2-AG, AEA, and cannabinoid CB1 and CB2 receptors may have medical benefits. First, the link between hippocampus mGluR activation and CB mobilization was strengthened in Fmr1-KO mice, while CB1R expression was unaffected (Zamberletti et al., 2017). In addition, an increase in striatal diacylglycerol lipase activity was observed (Maccarrone et al., 2010). Furthermore, the use of the drug JZL184 (which inhibits the breakdown of 2-AG by MAGL) to enhance 2-AG signaling corrected hyperactivity and anxiety in Fmr1-KO mice (Jung et al., 2012). In addition, it has been demonstrated that modulating AEA signaling can improve certain behavioral characteristics of Fmr1-deficient animals. In a study, a single injection of the FAAH blocker URB597 improved unpleasant memory and anxiety-like behavior in Fmr1-deficient mice without impairing their social behavior (Qin et al., 2015). In contrast, Wei et al*.* discovered that acute injection of URB597 to inhibit FAAH completely corrected the social deficit in Fmr1 deletion mice, indicating that boosting AEA activity at CB1 receptors may exhibit a prosocial behavior effect in mouse models of ASD (Wei et al., 2016). Furthermore, activation of either CB1 or CB2 receptors was found to alleviate certain behavioral symptoms of Fmr1-deficient animals. In the murine model, genetic and pharmacological inhibition of CB1 receptors with the CB1 receptor antagonist/inverse agonist rimonabant reversed cognitive impairments, epilepsy susceptibility, and nociceptive desensitization. Biochemically, CB1 receptor inhibition in the hippocampus of Fmr1 mutant mice corrected the overactivation of mTOR signaling and dendritic spine formation. Intriguingly, treatment with the CB2 inverse agonist AM630 had no effect on anxiety-like behavior or audiogenic seizure susceptibility, indicating that CB1 and CB2 receptors play distinct roles in the behavioral symptoms of FXS (Busquets-Garcia et al., 2013). A recent study by Gomis-Gonzales et al. validated the favorable effect of blocking CB1 receptors on the cognitive function of Fmr1 knockout mice. The authors demonstrated that low doses of rimonabant and the neutral antagonist NESS0327 prevented cognitive abnormalities in Fmr1 knockout mice, as determined by the novel object recognition test. Interestingly, the cognitive benefits of rimonabant were associated with the restoration of mGluR-LTD in the hippocampus of Fmr1-deficient animals (Gomis-González et al., 2016).

In addition, the VPA rat model has been widely used to assess the potential implications of the ECS in ASD. Rats treated with a single injection of VPA on gestational day 12.5 (GD 12.5) exhibited decreased mRNA expression of the enzyme primarily responsible for producing 2-AG DAGL, in the cerebellum, and enhanced activity of the 2-AG-catabolizing enzyme, MAGL in the hippocampus (Kerr et al., 2013). Gene expression of CB1 and CB2 receptors were unaffected; however, rats prenatally exposed to VPA showed altered expression of phosphorylated CB1 receptor in the amygdala, hippocampus, and dorsal striatum, with no alterations in the prefrontal cortex, cerebellum, and nucleus accumbens (Mangiatordi et al., 2023; Servadio et al., 2016). Modifications were also observed in the expression of other receptor targets for ECs, namely PPAR and GPR55 in the frontal cortex and PPAR and GPR55 in the hippocampus (Kerr et al., 2013). AEA and its congeners, oleoylethanolamide (OEA), and palmitoylethanolamide (PEA) were increased in the hippocampus of VPA-exposed rats immediately after social exposure, indicating that prenatal VPA exposure may have altered AEA signaling in response to interpersonal stimuli (Kerr et al., 2013). Rats exposed to VPA exhibited alterations in AEA metabolism from early life to adulthood. In fact, decreased expression of NAPE-PLD and increased expression of FAAH were observed in whole brains of rats treated with VPA (Servadio et al., 2016).

It is worth noting that increasing AEA signaling by inhibiting its degradation has been shown to alleviate the behavioral phenotype resulting from prenatal VPA exposure. Specifically, a systemic injection of the FAAH antagonist PF3845 at a dose of 10 mg/kg was found to ameliorate the social impairment observed in male mice exposed to VPA (Kerr et al., 2016). In comparison, PF3845 had no effect on the social behavior of female mice exposed to VPA, suggesting that FAAH inhibition may induce sexually dimorphic behaviors in VPA-exposed female mice (Kerr et al., 2016). Likewise, URB597 treatment improved the interaction problems of VPA-exposed pups in the homing test and reversed their social deficiencies in the three-chamber and social play behavior tests (Mechoulam, 2023; Servadio et al., 2016).

Moreover, recently Schiavi et al. examined the role of endocannabinoid neurotransmission in autism-like feautures in Fmr1-Δexon 8 rats. Their study revealed reduced anandamide in the hippocampus and elevated 2-arachidonoylglycerol (2-AG) in the amygdala of these rats. Increasing anandamide levels lessened cognitive abnormalities, while blocking amygdalar 2-AG transmission improved sociability in the rats, as demonstrated by behavioral tests (Schiavi et al., 2023). Considering the pivotal role of endocannabinoids in the etiopathology of ASD described in this article, it is not astonishing that researchers have investigated the therapeutic potential of certain phytocannabinoids, such as cannabidiol (Hill et al., 2023; Parrella et al., 2023; Shani Poleg et al., 2019). Although cannabidiol has only a weak affinity for CB1 receptors, it has been found to inhibit FAAH, which is responsible for the breakdown of AEA (Cristino et al., 2020; Parrella et al., 2023). This is thought to be particularly beneficial for individuals with ASD, who have been shown to have lower levels of AEA (Aishworiya et al., 2022; Aran et al., 2019). According to preliminary findings, cannabidiol has reduced symptoms of hyperactivity, self-injurious behaviors, anxiety, and sleep problems in ASD children. Recent clinical trials have not only demonstrated the effectiveness of cannabidiol in treating ASD symptoms, but also cognitive symptoms in individuals with FXS, without any adverse effects (Heussler et al., 2019; Tartaglia et al., 2019).

Although post-natal LPS injection is not widely accepted as a model of ASD, additional studies suggest that suppressing FAAH may be a treatment option for diseases characterized by reduced social behavior. In mice exposed postnatally to LPS, disruptions to the ECS have been described (Doenni et al., 2016; Mondal et al., 2023). Early-life inflammation caused by a single LPS administration on post-natal day (PND) 14 impaired both male and female adolescent social play and non-play behavior. Interpersonal impairments caused by LPS were associated with decreased CB1 receptor binding, higher AEA levels, and, interestingly, elevated FAAH activity in the amygdala. Prior to the social interaction test, oral administration of 1 mg/kg of the FAAH inhibitor PF-04457845 restored LPS-induced abnormalities in social behavior. A similar improvement was noticed following direct administration of PF-04457845 into the basolateral amygdala, suggesting that altered AEA signaling in this brain area play an important role in transmitting LPS-induced social deficits in at least female mice (Doenni et al., 2016; Shamabadi et al., 2024).

## Medical Cannabinoid and Risks

Agonists and antagonists of CB1 and CB2 have the potential to act as drug targets for ASD. However, there are several challenges in designing CB2 receptors-modulating drugs to alleviate ASD symptoms. CB2 receptors, like other lipid-binding receptors, bind to multiple non-specific ligands, making it difficult to design specific agonist and antagonist ligands for CB2 receptors due to off-target effects. The high abundance of CB1 receptors and other lipid-based endocannabinoid receptors also leads to more non-specific binding of CB2 ligands with CB1 receptors, triggering different downstream signaling pathways. Therefore, specific agonists or antagonists targeting CB2 receptors need to be synthesized and undergo multiple in vitro, in vivo, and clinical trials to minimize potential side effects (Atwood & Mackie, 2010). Different agonists of CB2 receptors can target other downstream regulatory molecules to modify their function. CB2 agonists display distinct of functional selectivity (Atwood et al., 2012a, 2012b; Mechoulam, 2023; Pinapati et al., 2024). For instance, aminoalkylindoles (e.g., WIN55,212-2, AM1241, etc.), which are common CB2 receptor agonists, cannot block calcium channels and do not have any role in CB2 receptor internalization. However, these molecules can activate the MAP kinase pathway and stimulate beta-arrestin2 (Shoemaker et al., 2005). They can recruit beta-arrestin2 to the plasma membrane and initiate different downstream signaling pathways. While blocking calcium channels and receptor internalization are crucial components of regulating any signaling pathway, aminoalkylindoles fail to do so (Nguyen et al., 2012). Therefore, standard CB2 receptor agonists can only control a portion of the signaling pathway. Similarly, inverse agonists and antagonists of CB2 receptors can also exhibit similar functional selectivity. Inverse agonists are drugs that selectively couple receptors to one type of downstream signaling molecule while reducing their association with other signaling molecules. Different inverse agonists can target different signaling molecules, so it is important to specify which molecules are being regulated and how they affect ASD before using these drugs as potential therapeutics. For example, commonly used CB2 receptor inverse agonists like SR144258 selectively target CB2 receptor internalization, whereas other like AM630 have a neutral effect (Atwood et al., 2012a, 2012b; Oka et al., 2005). It is important to specify the regulatory molecules and their impact on ASD for every agonist and antagonist before using them as potential therapeutics. Further research, animal trials and clinical trials are needed to focus on these areas. Furthermore, functional efficacy is also an essential parameter for the application of any drugs, as the density of receptors and downstream signaling molecules determine the practical impact of ligands. Moreover, functional efficacy varies in different cellular and physiological conditions in GPCR targeting drugs (Efron & Taylor, 2023; Strange, 2002). In the case of in vitro studies, transfected cells of CB2 receptors are used to evaluate the functional efficacy of ligands. However, the density of CB2 receptors is usually low in physiological conditions. Therefore, it is important to note that any agonists that work effectively in vitro may only act as partial agonists in physiological conditions. On the other hand, antagonists may behave as a reverse agonists in different physiological conditions, which could be additional challenge to the effective function of drugs particularly in cases where there is a higher density of receptors or downstream signaling molecules (Fong & Heymsfield, 2009). However, the likelihood of this problem occurring in CB2 receptor antagonists is minimal due to their low physiological density. While drugs targeting CB1 receptors are commonly used to treat different brain pathophysiological diseases, drugs targeting CB2 receptors may have a less significant off-target effect due to their low density in normal physiological conditions. Beside this, using cannabinoid-based drugs to treat children and adolescent patients affected by ASD has raised many social and legal controversies (Bou Khalil, 2012). During childhood and adolescence, several critical brain development processes occur, and it is believed that cannabinoid-based drugs may have adverse effects on the brain (Poleg et al., 2019) or induce cannabis addiction. Some clinical data also support these arguments (Bailone et al., 2022; Parrella et al., 2023; Shani Poleg et al., 2019; Salloum-Asfar et al., 2023). For instance, a preclinical rodent study found that chronic application of CB2 receptors agonist WIN 55,212-2 during puberty resulted in severe behavioral disturbance in adulthood (Schneider & Koch, 2003; Schneider et al., 2008).

From the cell biological and neuroimmunological perspective, targeting CB2 receptors with agonists and antagonists could offer new therapeutic options for ameliorating ASD-related symptoms. Promising results have been observed in preclinical and in vitro studies. However, the available clinical trials data is disappointing, with many drugs being withdrawn or failing to reach their primary pharmacological targets. This disparity between preclinical and clinical data highlights the need for further pharmacological studies to determine the clinical and functional efficacy of CB2 receptor-based drugs. To develop possible drugs, humanoid rodent models or 3D organoid models could be used for in vitro studies, while considering all aspects of CB2 receptor pharmacological properties. With careful research and development, CB2-based therapeutics may soon become available in clinics, providing a potential cure for ASD patients.

## Conclusion & Perspectives

Clinical and preclinical evidence clearly supports the role of the ECS in the etiopathogenesis of ASD and its potential for medication development. According to Geschwind’s “many genes, similar pathways” concept (Geschwind, 2008), evidence from ASD-related mouse lines and pharmacological interventions targeting the ECS in wild-type animals suggests that an imbalance in ECS signaling is a possible common etiopathological route of this complex condition. This is consistent with the ECS's substantial modulatory effect on neural functioning and cognitive maturation. However, this field remains unexplored, and many researchers have emphasized the need to understand how ECS failure contributes to aberrant brain maturation. The use of neurons derived from induced pluripotent stem cells is anticipated to provide new insights, as it has already contributed to our understanding of the etiology of various neuropsychiatric disorders, in order to distinguish the significance of ECS in abnormal brain growth from fully developed synaptic functionality (Wen et al., 2014; Yeh & Levine, 2017).

Although preclinical data suggest that modifying the ECS through the pharmaceutical therapies may be useful for alleviating ASD symptoms, no definitive conclusions can be drawn due to the early stage of research. Evidence suggests that increasing AEA signaling by inhibiting its disintegration promotes prosocial behavior in several mouse models of ASD. Furthermore, acute or chronic inhibition of CB1 receptors has been shown to have positive effects on cognitive impairments in animal models of FXS. Interestingly, medication was systematically administered in most investigations. However, changes in the ECS documented in mouse models of ASD seem to vary depending on the specific brain area studied, suggesting a potential diverse contribution to ASD-like manifestations. If so, it is unlikely that any prospective treatment method would rely on a single targeted molecule.

In this review, we aim to demonstrate that the ECS’s role in ASD is a near foregone conclusion, based on the vast amount of data presented here. However, we do not intend to imply that the ECS alone can explain the etiopathology of ASD. On the contrary, we, along with other experts in the field, are convinced that any comprehensive understanding of ASD must incorporate parallel pathogenic elements. This complex neurodevelopmental disorder is essentially caused by intricate interactions between parallel systems that regulate brain development. The ECS may provide a clue to the identity other major players and the complexity of the situation. Accordingly, drug design should seek new molecular pathways for multi-target pharmacology.

## Data Availability

No datasets were generated or analyzed during the current study.

## Abbreviations

2-AG: 2-Arachidonoylglycerol
3D: Three dimensional
AEA: Anandamide
ASD: Autism spectrum disorder
ATP: Adenosine triphosphate
BDNF: Brain-derived neurotrophic factor
cDNA: Complementary DNA
CNR2: CB2 receptor genes
CNS: Central nervous system
DAGL-α: Diacylglycerol lipase alpha
eCBs: Endocannabinoids
ECS: Endocannabinoid system
FAAH: Fatty acid amide hydrolase
FXS: Fragile X syndrome
GPR55: G-protein-coupled receptor 55
IL-1: Interleukin 1
IL-12: Interleukin 12
IL-17: Interleukin 17
IL-1β: Interleukin 1β
IL-6: Interleukin 6
LPS: Lipopolysaccharide
MAGL: Monoacyl-glycerol lipase
MECP-2: Methyl-CpG binding protein 2
mGluR-LTD: Group I metabotropic glutamate receptor-dependent long-term depression
MIF: Macrophage inhibitory factor
mRNA: Messenger RNA
mTOR: Mammalian target of rapamycin
NAPE-PLD: N-acyl phosphatidylethanolamine-specific phospholipase D
NK cells: Natural killer cells
NLGNs: Neuroligins
OEA: Oleoylethanolamide
PCR: Polymerase chain reaction
PEA: Palmitoylethanolamide
PET: Position emission tomography
PND: Post-natal day
PNS: Peripheral nervous system
PPAR: Peroxisome proliferator-activated receptors
RTT: Rett syndrome
TGF-β: Transforming growth factor beta
THC: 9-Tetrahydrocannabinol
THIK-1: Tandem-pore domain halothane-inhibited K^+^ channel 1
TLR: Toll-like receptor
TNF-α: Tumor necrosis factor-alpha
VPA: Valproic acid
